# Combating illicit fentanyl: Will increased Chinese regulation generate a public health crisis in India?

**DOI:** 10.3389/fpubh.2022.969395

**Published:** 2022-10-14

**Authors:** Chao Wang, Nicholas Lassi

**Affiliations:** Guanghua Law School, Zhejiang University, Hangzhou, China

**Keywords:** India, China, fentanyl, class-wide scheduling, global drug governance, drugs, public health, synthetic opioids

## Abstract

This study examines how Chinese drug regulations are shifting illicit fentanyl production from China to India. This change has implications for the law, drug enforcement, pharmaceutical industry, and public health, as domestic production increases domestic access to fentanyl, in India. An empirical examination of current trends in fentanyl production and use in the region is conducted, along with an exegesis of the legal and regulatory systems in China and India. There is an accounting of the chemical and pharmaceutical industries, the strengths and weaknesses of drug legislation, and the public health consequences of increased production, distribution, and use of fentanyl in India. This study also details how the Indian government meets this challenge through legislative mechanisms, foremost through class-wide legislative control over fentanyl and its precursors. Class-wide control prohibits the unlawful production and distribution of all current and future fentanyl analogs and their precursors, disincentivizing and disrupting their development and production. The Indian government should also reduce domestic demand by promoting harm reduction measures such as opioid substitution therapy, evidence-based treatment, fentanyl test strip and naloxone distribution, and needle exchange programs.

## Introduction

In 2018, two Indian nationals and a Mexican collaborator were arrested in the Indian city of Indore in possession of around 10 kg of illicitly manufactured fentanyl (IMF). At the time of the arrest, the three men were wearing protective gear used for handling dangerous chemicals, and it is alleged that they were conspiring to transport the IMF to Mexico (1). The IMF was apparently manufactured in a factory operated by one of the Indian nationals using the popular fentanyl precursors NPP [1-(2-phenylethyl) piperidin-4-one] and ANPP [N-Phenyl-1-(2-phenylethyl) piperidin-4-amine] (2). This same factory also manufactured many legal substances and scientific products (3), showing the difficulty in regulating these primarily legal operations moonlighting in illegality. The Mexican collaborator was reputedly associated with the Sinaloa cartel, indicating that Mexican cartels and transnational criminal organizations (TCO) are working closely with Indian laboratories to produce and distribute IMF. This specific operation was previously performed in China, but increased regulations have made obtaining NPP, ANPP, and other fentanyl precursors in China troublesome, prompting its relocation to India (4).

Also, in 2018, four Indian nationals were arrested in Mumbai with around 100 kg of NPP, the aforementioned precursor synthesized into fentanyl using the Siegfried method (2)^1^. The NPP was purportedly being transported to Mexico, where, after undergoing two reactions, it would become the immediate fentanyl precursor ANPP, which is one simple reaction away from producing fentanyl. Once synthesized into fentanyl, it was to be smuggled into the United States (5). The NPP, as is almost universally the case with IMF and fentanyl related substances (FRS), was mislabeled and mis-declared as a licit chemical for its international transportation. According to the DEA (2), this was the third time in 2018 that IMF or FRS was confiscated in India with links to Mexican TCOs.

IMF and other synthetic opioids are commonly found among the thousands of parcels containing illicit narcotics mailed from India and intercepted by the U.S. government every year (4, 6). Numerous business-to-business (B-2-B) and commercial websites registered and operating in India currently market and distribute IMF and FRS for international buyers (6). India is also a major manufacturer of illicit precursors in general. According to the U.S. Department of State, “Multi-ton shipments of (illicit) precursor materials from India have been shipped to Africa and Mexico” [(6); 64].

Indian chemists have a long history of manufacturing and distributing large quantities of the synthetic opioid tramadol for global consumption and global diversion. Prior to India's 2018 scheduling of tramadol, most of the confiscated tramadol in West Africa was manufactured in India (7). For example, in 2017, authorities in Ghana confiscated 524,000 tramadol tablets, of which 87% had dosages exceeding those commonly produced for medical purposes. Most of this tramadol originated in India (8). In 2018, the Indian government amended the Narcotic Drugs and Psychotropic Substances Act, 1985 (9), to control tramadol, placing it under the supervision of the Narcotics Control Bureau (NCB), and increasing punishments for its unlawful production and distribution (10). This legislation has reduced tramadol's global availability, but significant amounts are still illicitly distributed by Indian manufacturers. The Indian government confirmed to the UNODC that at least five shipments of tramadol originating in India were stopped from late 2018 to 2019 due to fabricated import documentation. The destinations for these shipments include Afghanistan, Benin, Nigeria (25 kg), and two shipments to Somalia (7).

The increased production of IMF and FRS in India is largely the result of increased Chinese regulation. A 2020 DEA report provides insight, “The India- and China-based suspects (in the Indore case) shifted their production from China to India, likely due in part to China's regulation of ANPP and NPP. The organization likely transferred their production to India due to difficulties obtaining precursor chemicals in China and the increasing pressure from Chinese authorities on fentanyl manufacturing operations” (2, 4). That Chinese manufacturers and TCO's would shift IMF production from China to India is understandable, as India has a large population of highly trained chemists, a strong chemical and pharmaceutical infrastructure, a large total population that is difficult to monitor, weak fentanyl regulations, high-levels of poverty, and corrupt local officials (6). Few places in the world possess this mixture of know-how, infrastructure, weak legislation, poverty, and local corruption. Socioeconomic factors, namely poverty, corruption, and limited access to education, have been linked to illicit drug production and distribution in locations across the globe. For example, poverty in Myanmar and Pakistan has historically been linked to illicit narcotics production (11–13).

This study analyzes how improved Chinese regulation of IMF and FRS is shifting production from China to India (2), and how this relocation has ramifications for the law, chemical and pharmaceutical regulation, narcotics enforcement, and public health in India. Further, how India meets this challenge through legislative channels is detailed, specifically the necessity for class-wide legislative control over fentanyl and its precursors. Proposals for refining existing laws and regulations, along with the means to reduce negative public health outcomes, are extended.

## Assessment of policy/guidelines options and implications

An empirical investigation into the current trends in fentanyl production and use in India, along with an assessment of the legal and regulatory regimes in China and India, is forwarded. A logic model approach is deployed, wherein the key inputs, processes, responses, and effects of intensified Chinese fentanyl regulation is established, and this is put into the context of fentanyl production, distribution, use, and regional public health within India. To this end, fentanyl legislation was analyzed from numerous sources, including Chinalawinfo.com, the central database of laws and cases in the PRC, LexisNexis India, the Indian Ministry of Finance (Department of Revenue), and the Narcotics Control Bureau (NCB), an official source of current Indian drug law. Additional legal resources and announcements were examined, specifically PRC communiqués, The Gazette of India, which publishes official legislative announcements from India, and the U.N. Office on Drugs and Crime (UNODC). Reports from a variety of regulatory bodies were also reviewed, such as those from the State Council of the PRC, Ministry of Home Affairs of the Indian Government, International Narcotics Control Board (INCB), and the U.S. Drug Enforcement Administration (DEA). These resources were chosen based on their import, reliability, date of publication, and aptness in covering legal and social issues involving fentanyl law.

## Background

Fentanyl is up to 50 times more potent than heroin and up to 100 times more potent than methadone (14). Potency increases among some of fentanyl's more dangerous and increasingly popular analogs. For example, methylfentanyl is up to 15 times more potent than fentanyl, and carfentanil, first used by veterinarians to tranquilize large mammals, is around 100 times more potent than fentanyl (15, 16). These synthetic opioids and similar others are the cause for shocking levels of overdose deaths in the U.S. and around the world (17). The single greatest cause of death for people between the ages of 18 and 45 in the U.S. is overdosing on synthetic opioids (18). The U.S. experienced a record 100,306 overdose fatalities in the 12-months spanning May 2020–April 2021, rising 28.5% from the prior year (19). Around 64% of these fatalities (20), about 150 overdose deaths per day (21), were linked to synthetic opioids (non-methadone).

National surveys by the Indian Ministry of Social Justice and Empowerment detail a striking rise in illicit opioid use among Indian males. A national survey taken in 2004 found that 0.7% of Indian males used illicit opioids; the same survey collected in 2018 found a rise of over 3%, with 3.97% active illicit opioid users (22). Even though some of this difference may be due to methodological improvements in survey distribution, the general outlook should be troubling for even passive observers. India has around double the rate of illicit opioid use compared to the worldwide average and around quadruple that of the Asian average. The UNODC (23) estimates that 0.7% of the world's population use opioids, and 0.46% of people in Asia use opioids; Ambekar et al. (22) estimates that around 2.06% of the Indian population consume opioids (either pharmaceutical opioids, opium, or heroin), with ~0.7% of users in need of addiction treatment. These opioid users generally favor heroin, but heroin is often in short supply and costly, so they turn to roadside pharmacies for cheaper and less potent opioids like tramadol (24, 25). An influx of cheap and highly potent IMF, resulting from increased domestic production, has and will continue to alter this dynamic. Why mess with unpredictable and costly heroin or less potent corner store opioids like tramadol when IMF is available. This reasoning has taken hold in the U.S. (26), and, without serious legislative and enforcement control, will continue to manifest in India as well.

## Licit and illicit opioid production and distribution in India

India has a 42 billion-dollar domestic pharmaceutical industry, with much of the production centered in and around the Hyderabad area (27). This figure is expected to grow to 65 billion by 2024 and 120–130 billion by 2030 (28). India's pharmaceutical industry ranks third in the world in output by volume, with an estimated 3,000 pharmaceutical companies and over 10,000 production facilities (29). It also supplies around 20% of the world's demand for generic drugs (28). The passage of The Patents Act, 1970, dropped composition of matter patents for many drugs, allowing Indian chemists to reverse-engineer and produce industrial level quantities of numerous generic drugs, effectuating the exceptional growth of India's pharmaceutical industry (30).

The Indian government has recently invested heavily in health care, health insurance for the impoverished, and over 150,000 new government run primary care facilities, which are expected to open in the early 2020's (31). The introduction of COVID-19 has accelerated government expenditures on health care, with health spending increasing almost 73% from 2019–2020 to 2020–2021 (32). The Indian government is also pushing to reduce India's dependency on chemicals and pharmaceuticals imported from China (33). The supply chain slowdown during the COVID-19 epidemic, which disrupted the production and distribution of Chinese pharmaceuticals and precursor chemicals (34), has motivated the Indian government to reduce imports from a country that is generally more competitor than friend and engagements in border skirmishes, that could develop into larger conflicts, are common.

For several decades the Indian government prohibited the Indian people from consuming opioids for medicinal pain management under its drug and pharmaceutical laws, despite India being a major manufacturer of medicinal opioids, particularly fentanyl and tramadol, for export and global consumption (35). In 2014, after years of lobbying by palliative care experts, the Indian government amended the NDPS Act to allow citizens access to medicinal opioids, acknowledging that the government was responsible for managing pain (36). This amendment, along with large injections of government funding for pain management medicine and clinics, significantly altered India's domestic pharmaceutical, pain management, and opioid industries.

Most large Indian hospitals now have wings designated for pain management, and private, for-profit, pain management clinics are proliferating (25, 37). Also, many pain management substances are easily purchased at small corner store pharmacies referred to as chemists. The pain management industry has become highly profitable for many companies and businesses, and the Indian population is now inundated with opioids and misuse and addiction are common. Over-prescription by inexpert medical practitioners and the inadequate regulation of roadside pharmacies are founts of misuse and abuse. With millions of doctors and pharmacies covering India's vast, poorly monitored, and often chaotic medical system, the regulation and supervision of the distribution of opioids is limited.

To obtain licensing, large Indian hospitals are now required by their professional licensers to evaluate pain as one of five vital signs (37). Pain has joined body temperature, pulse, blood pressure, and respiratory functioning in the standard evaluation of patients. Overemphasizing pain, designating it a fifth vital sign, promoting both public and private pain management clinics, loose distribution oversight, and normalizing the consumption of pain medicines to resolve numerous medical problems, has played out in the U.S. over the last few decades as well, precipitating the prescription opioid epidemic in the U.S. from the mid-1990's to the 2000's, and igniting the current fentanyl crisis (38).

This creates an environment where people become addicted to opioids through legal and quasilegal pain management channels. Once dependent and less capable of affording strong medicinal opioids, or losing the capacity to legally obtain these opioids, people will then switch to cheap and potent IMF or its analogs. Increased Indian opioid addiction, combined with an upsurge of IMF production in India, portends a major Indian public health crisis. Ambekar et al. (22) describes how this concern is building, “In as many as 13 states in the country (India), the prevalence of opioid use disorders is more than one percent (indicating a major public health concern)” (37).

India currently possesses the chemical and pharmaceutical infrastructure necessary to significantly increase IMF and FRS production (28). Indian chemists typically manufacture illegal chemicals in disused laboratories located in unpopulated areas (39). IMF's low risk of detection during production, ease of manufacture, cheap production costs, and high potency to weight ratio all represent enticing incentives for chemists and manufacturers. A new batch of fentanyl can be produced in several days by a few competent chemists in a small laboratory. Papaver somniferum (opium poppy), on the other hand, requires a 3-month growth period, significant land for cultivation, armed guards for crop protection, and further processing. Additionally, satellite technology, deep learning technology (40), unmanned aerial vehicle (UAV) surveillance (41), and other advanced methods used to locate opium growth sites have made illicit opium cultivation increasingly risky for drug syndicates worldwide (42).

## The shift in IMF and FRS production from China to India

Bad actors in China have recently experienced disruptions in their ability to produce and distribute completed fentanyl and its analogs (43, 44). For example, in 2018, the U.S. reported 314 cases of confiscated fentanyl transported directly from China, with an overall weight of 278.34 pounds, while in 2019, the year that China enacted class-wide control over fentanyl (43), the U.S. reported only 12 cases of confiscated fentanyl from China, with an overall weight of 11.58 pounds (45). Bad actors in China are experiencing less disruption in the production of fentanyl precursors (2). The key for Chinese manufacturers and distributers is that the final batch of fentanyl is produced off Chinese soil to maintain a thin vail of legality. This method has been effective. Many Chinese chemical and pharmaceutical companies have gotten rich by manufacturing fentanyl precursors and precursor analogs and distributing them to international locations (44), namely Mexico, and now India, to be synthesized into fentanyl. Once fentanyl is produced, it is generally transported into the U.S. for distribution and sale.

Controlling the production of fentanyl and its analogs is more straightforward, as laboratories are either producing an obviously illicit substance meeting molecular requirements and psychoactive effects or they are not, than it is controlling the production of fentanyl precursors. Over 5,000 Chinese pharmaceutical companies are producing thousands of pharmaceutical products in a fluid environment of new synthetics (46). Many of the precursors used to manufacture fentanyl are also used to manufacture legal substances, and most fentanyl precursors don't produce any meaningful psychoactive effects until synthesized into fentanyl. Because these precursors are usually one to five steps away from fentanyl in the production process, a large gray area exists where bad acting can flourish. Additionally, the massive and somewhat chaotic nature of the Chinese chemical industry makes it easy for chemical companies to set up front operations wherein inspectors are shown only a portion of the operations, and the rest, the illicit or questionable operations (e.g., producing legal, though obviously troubling and internationally condemned, fentanyl precursors) are hidden in other areas or other industrial parks (44). These issues and tactics all coalesce to place government inspectors and regulators at a serious disadvantage when attempting to control or even document the problem.

Though, to shield against being labeled a narco-state, the Chinese central government has been ramping up efforts to reduce China's production of illicit fentanyl precursors and their analogs. Chinese manufacturers and distributers can see the writing on the wall and are slowly shifting the production of IMF and FRS to India (2).

Significant legal and institutional changes in India are necessary to control this problem. It is easy for states to disregard the manufacture of illicit narcotics if the drugs are distributed to foreign lands for consumption (even better if the illicit drugs are consumed by competitors or unfriendly states). For example, for over two decades Chinese laboratories produced and distributed methamphetamines and fentanyl for illicit consumption overseas, largely to the U.S. (2, 47, 48), generally sparing the local population from the destruction caused by these substances. India will have more difficulties confining these substances to non-domestic distribution. Comparing India to China in government structure, history with illicit substances, identification with the West, and the liberal/conservative divide, indicates that IMF and FRS will pose a more significant public health threat to Indian society than it does to Chinese society. China has a strong central government with a great capacity to enact controls over its citizens when necessary. Opium use was mostly eradicated under Chairman Mao Zedong's anti-opium campaigns in the early 1950's (49), and the outcome of the opium wars and the historical effects of opium still resonate with Chinese today, rendering opioids culturally unpopular. Conservative Confucian ethics also remain powerful within modern China (50); a cultural system categorically opposed to drug use and drug culture. India has a democratic government with a limited ability to influence the lives of its citizens. India is more liberal (51), with views aligning more with the West than with Confucian conservatism. India also has a long history of opium consumption and opium acceptance (52), providing fertile ground for this synthetic relative of a somewhat tolerated substance to overwhelm India's public health system. There is a high probability that IMF and FRS will be produced in India and consumed in large quantities there as well.

## The strengths and weaknesses of Indian narcotics legislation

The Narcotic Drugs and Psychotropic Substances Act (9) of 1985, located in article 47 of the Indian Constitution, is legislation to “Consolidate and amend the law relating to narcotic drugs, to make stringent provisions for the control and regulation of operations relating to narcotic drugs and psychotropic substances” (9). This Act applies to all Indian citizens inside and outside of India, and has been amended four times, in 1988, 2001, 2014, and 2021. The Narcotics Control Bureau (NCB) operates at the behest of the Central Government to enforce the NDPS Act (53). The NCB coordinates different government offices [Central Economic Intelligence Bureau (CEIB), Central Bureau of Investigation (CBI), state police, customs, and other agencies at the national and state levels] to combat the manufacture, distribution, and use of illicit drugs domestically and internationally. The NCB enforces the conditions of the NDPS Act through the Prevention of Illicit Trafficking in Narcotic Drugs and Psychotropic Substances Act (1988), gathers intelligence on illicit drug operations, inspects India's borders for smuggling activities, and ensures that India is conforming to its international treaty obligations, namely the three U.N. international agreements shaping India's domestic drug control policy: the Single Convention on Narcotic Drugs of 1961 (54), which scheduled fentanyl and other synthetic substances, the Convention on Psychotropic Substances of 1971 (55), and the United Nations Convention against Illicit Traffic in Narcotic Drugs and Psychotropic Substances of 1988 (56), which controlled precursors employed in the manufacture of illicit substances.

India has a history of leniency on illicit opioids, going so far as to make serious inroads toward the legalization of opium in 2015. Patiala MP Dr. Dharamvira Gandhi authored legislation seeking to decriminalize opium through an amendment in the NDPS Act. Although this legislation was an effort to reduce the overall use of opioids, diminish the influence of mafias and other dangerous actors, and “provide relief to common drug user (sic) through cheap, regulated and medically supervised supply of traditional and natural intoxicants like “afeem” and “bhukki” (opium) “to get society rid of dangerous and killing medical and synthetic drugs”” (57), it arguably demonstrates an openness toward opium that is seldom displayed in much of the world. Ambekar et al. (22) explains how Indian culture generally tolerates opium, “Opium…enjoys socio-cultural acceptance in many parts of the country…” (42).

India only controls a fraction of the FRS controlled in China (58), and has not banned well-known FRS that are banned in the PRC, U.S., and in many other countries (9, 43, 59, 60). Westhoff (44) explains, “Whereas China has been at least somewhat responsive to American requests to control its chemical industry, India has trailed when it comes to scheduling NPS (new psychoactive substances) and fentanyl precursors” (225). It seems as if the Indian government's interest in keeping pace with new FRS has stalled, despite a rising number of novel fentanyl analogs and their precursors (61), and an increase in the production of IMF and FRS in India. There is little expectation that India, provided its recent history, will keep up with China in scheduling FRS. This communicates to local Indian chemists and their Chinese collaborators that the Indian legal and regulatory regimes are either unconcerned or too gridlocked to legislate and control FRS.

In 2018, both China and India banned the popular fentanyl precursors NPP and ANPP (62, 63). As stated in the Indian Ministry of Finance (Department of Revenue) notification, “This order may be called the Narcotic Drugs and Psychotropic Substances (Regulation of Controlled Substances) Amendment Order, 2018…the following numbers and entries shall be inserted, namely: “18. 4-Anilino-N-phenethylpiperidine (ANPP), 19. N-Phenethyl-4-piperidone (NPP)”” (1, 63). Though, unlike China, Indian enforcement is weak, which is a major reason that NPP and ANPP are becoming more prevalent in India.

In 2019, China enacted class-wide scheduling over all fentanyl analogs (58), signaling increased control over the production and distribution of synthetic opioids. India has not enacted such legislation for fentanyl analogs. The Chinese class-wide ban over fentanyl analogs provides an example of the legislation India should pursue to control fentanyl analogs, and, taking this a necessary step further, the type of ban that should be established for fentanyl precursors as well. To counter the growing number of fentanyl analogs being manufactured in the PRC, the Chinese government enacted class-wide control over all current and future fentanyl analogs (58). The State Council (43) of the PRC explains:

The Ministry of Public Security, the National Health Commission, and the National Medical Products Administration (NMPA) decided to list fentanyl-like substances. Included in the supplementary list of controlled varieties of non-medical narcotic drugs and psychotropic substances. “Fentanyl-like substances” refer to substances whose chemical structure is compared with that of fentanyl and meets one or more of the following conditions:

- Replacement of the N-propionyl group by another acyl group;
- Replacement of the N-phenyl group with any aromatic monocycle whether or not further substituted in or on the aromatic monocycle;
- Substitution in or on the piperidine ring with alkyl, alkenyl, alkoxyl, ester, ether, hydroxyl, halo, haloalkyl, amino or nitro groups;
- Replacement of the phenethyl group with another group, excluding hydrogen atom [Translation by the (58)].

The U.S. has also legislated class-wide control over fentanyl analogs. The Controlled Substance Analog Enforcement Act of 1986 (otherwise known as the Federal Analog Act, 21 U.S.C. § 813) categorized any substance closely related to a Schedule I or II substance, in both chemical structure and psychoactive effect (or expected psychoactive effect), as Schedule I. Any substance closely related to a scheduled substance becomes illegal at conception, including precursors if certain conditions are satisfied [being an immediate precursor to a scheduled substance is the main condition; immediate fentanyl precursors automatically fall under Schedule II control, 21 U.S.C. 811(e)]. If chemical manufacturers or distributers in the U.S. know, or reasonability should know, that they are producing or distributing substances chemically analogous to scheduled substances with substantially similar psychoactive effects, they are subject to legal consequences corresponding to illegally producing or distributing a Schedule I substance. Without a class-wide ban on fentanyl and its precursors, Indian chemists can legally manufacture many fentanyl precursors and their analogs and then legally manufacture many fentanyl analogs from these precursors.

The Indian government prohibits substances through statutes in the NDPS Act. The principle provision from which substances are prohibited is found in Chapter III, part 8, which reads:

Prohibition of certain operations. —No person shall—

- cultivate any coca plant or gather any portion of coca plant; or
- cultivate the opium poppy or any cannabis plant; or
- produce, manufacture, possess, sell, purchase, transport, warehouse, use, consume, import inter-State, export inter-State, import into India, export from India or transship any narcotic drug or psychotropic substance.

Except for medical or scientific purposes…by way of license, permit or authorization also in accordance with the terms and conditions of such license, permit or authorization (9).

It is clear, under this provision, that only certain narcotic drugs themselves, and not their analogs, or other substances similar in chemical makeup or psychoactive effect, are prohibited. This leaves ample room for Indian chemists to legally manufacture unscheduled fentanyl analogs and their precursors. Chemists simply need to make small molecular adjustments to the chemical structure of fentanyl to produce legal analogs, which can reach levels of potency and danger equal or greater to fentanyl itself.

Substances are incorporated into Indian law individually by the Central Government once they are proven, through persuasive evidence, to be of the nature and effect, along with abuse potential, of a narcotic substance. The guidelines for adding or removing substances in the NDPS Act as stated in Chapter I, part 3:

Power to add to or omit from the list of psychotropic substances. —The Central Government may, if satisfied that it is necessary or expedient so to do on the basis of—

- the information and evidence which has become available to it with respect to the nature and effects of, and the abuse or the scope for abuse of, any substance (natural or synthetic) or natural material or any salt or preparation of such substance or material; and
- the modification or provisions (if any) which have been made to, or in, any International Convention with respect to such substance, natural material or salt or preparation of such substance or material.

By notification in the Official Gazette, add to, or, as the case may be, omit from, the list of psychotropic substances specified in the Schedule (sic) such substance or natural material or salt or preparation of such substance or material (9).

Fentanyl analogs and their precursors are not immediately and automatically added to the list of banned substances in the NDPS Act, as they would under class-wide control. India should institute a class-wide ban on fentanyl and its precursors as a message to the PRC, U.S., and the international community that they are serious about stemming the manufacture and distribution of these substances at the outset (before a major Indian public health crisis or before India takes on the narco-state label). This legislation will likely impede the production and distribution of these substances by nefarious chemical companies and independent manufacturers, as similar legislation has done for fentanyl analog production in China (14). It will also disincentivize Chinese cartels and other Chinese parties manufacturing IMF and FRS from cooperating with Indian laboratories.

## The public health consequences of increased production of IMF and FRS in India

Stricter Chinese drug regulation has increased Indian IMF and FRS production (2). This shift has the potential to generate a significant Indian public health crisis, as domestic production pushes more product into local areas, increasing use, addiction, crime, and overdose deaths (64). India possesses limited harm reduction channels (65), such as treatment programs, opioid substitution therapy (OST), overdose reversal antidotes, and emergency services for those overdosing, to insulate against a large-scale public health crisis.

Of all illicit narcotics consumed in India, opioids are perennially the majority cause of years of life lost due to overdose deaths and drug related unemployment (66). IMF can be injected intravenously both independently and mixed with heroin and other narcotics to boost potency. Intravenous drug use, and its accompanying needle sharing, increases the transmission of HIV and other blood-borne diseases, such as hepatitis B (HBV) and hepatitis C (HCV). A rise in intravenous drug use in certain geographic locations often corresponds with increased blood-borne diseases in those same locations (67).

To get a sense of the transmissibility of HIV and HCV among people who inject drugs (PWID), Kaplan and Heimer (68) examined the pervasiveness of needles infected with HIV in a New Haven, Connecticut, government authorized needle exchange program. In their sample of used needles in this exchange program, 67.5% tested positive for HIV. Two studies conducted in New Zealand found high rates of HCV antibodies among PWID. Kemp et al. (69) determined that 64% of PWID tested positive for HCV antibodies, and Robinson et al. (70) found that 77% tested positive for HCV antibodies. High rates of infection from needle sharing in developed countries, with regular needle access and harm reduction programs, indicates that India, a developing country where users have limited needle access and harm reduction programs, will face serious public health and economic disruptions from IMF and FRS.

India has an estimated 1.7 million PWID, with an estimated 2.2 million people infected with HIV (71), figures that will likely rise as significant IMF and FRS use takes hold. HIV infection is high and increasing among PWID in India. Lucas et al. (72) found that among active PWID in India, ~18.1% were HIV positive, needle sharing was common, and women were three times as likely to have HIV. McFall et al. (73) found that female intravenous drug users in India accounted for 15.9% of users, and among these female users, 52.9% were infected with HIV and 22.3% had HCV. Among the men, 17.4% had HIV and 30.4% had HCV. Clipman et al.'s (74) study found even higher rates among Indian men using intravenously, with 37% infected with HIV and 46.8% infected with HCV. Intravenous drug use is the engine behind the HIV crisis in northeastern India, an area largely underdeveloped and geographically linked to the Golden Triangle region (22). This area, replete with heavy drug use, including heroin, opium, and synthetic opioids (75), will likely be an epicenter of IMF and FRS use in the future and is a tinderbox for a significant public health crisis.

Heroin, MDMA, methamphetamine, and other hard narcotics are commonly cut with fentanyl to increase their potency. Chinese polydrug users who combined heroin and synthetic drugs (SD) (mostly IMF), when compared to either exclusive heroin or SD use, exhibited more frequent drug use, higher drug sharing (among friends and at gatherings), and riskier sexual behavior (76). Chinese PWID who combined heroin and SD were also more likely to have HIV (10.5%) and syphilis (3.6%) compared to single drug users. Su et al.'s (77) modeling of drug use patterns in China predicts this trend to continue until at least 2035, during which polydrug use will produce the most HIV and HCV infections among users. The infusion of fentanyl into the Indian drug supply will likely produce similar results. Interestingly, Chinese PWID who used SD exclusively engaged in more frequent and risky sexual activity relative to polydrug or heroin-only users (78). If IMF increases sexual activity, increased use in India may contribute to the rise of HIV, HCV, and other sexually transmitted diseases.

Younger PWID in India engage in more dangerous drug and sex related activities. Young-adult male PWID in India, compared to older adult male PWID, are more likely to engage in needle sharing, have unprotected sex^2^, and have more sexual partners (80). Young-adult males are also less likely to test for HIV and less likely to partake in harm reduction programs. Increased needle sharing, unprotected sex, and multiple partners, along with the introduction of cheap, potent, and deadly fentanyl, offers several avenues for mortality, social and economic disruption, and, taken together, a major public health crisis in India.

High rates of synthetic opioid use produce significant losses in economic productivity (81). HIV, HCV, and sepsis, all prevalent among PWID, exert a major financial strain on global health care systems (82). Sepsis, a complication caused by bacterial infections, often results from unhygienic needle use, and, left untreated, has a high mortality rate. Extended time in the ICU and premature death, resulting from severe sepsis, exerts considerable economic losses (83, 84). PWID are also more likely to be homeless or experience unstable living conditions (85). Female intravenous drug users in India are particularly impacted by housing and other living insecurities (86). Increased IMF and FRS use in India will have detrimental consequences for all levels of society, from common drug users to the politicians responsible for social stability and economic output.

A problem in determining the level of fentanyl use in India is that drug use information, specifically regarding overdoses, is ineffectively tracked and collected (64). Users who overdose on drugs are rarely tested to determine the exact cause of overdose, which makes an appraisal of the influence of IMF or FRS in a regional drug supply difficult. What is considered a string of heroin or methamphetamine overdoses may be the result of IMF or FRS cut into the drug supply, and this may go unnoticed. The reach and penetration of fentanyl into many regions in India may be difficult to determine until extended time has passed, serious testing has been conducted, and significant damage has been done. Therefore, it is important for law enforcement to employ fentanyl test strips (FTS) at overdose sites to quickly pinpoint fentanyl hotspots. When fentanyl is detected, more attention and harm reduction resources can be provided to those areas (87).

## Criticisms of blanket ban legislation

India should enact class-wide control over fentanyl and its precursors. Though, there are concerns that class-wide bans stifle necessary scientific research, as each fentanyl analog and precursor analog would require an individual permit from a governing body (entities often slow to act and hold the potential for corruption). Indian law has legislation in place that can be transferred to suit class-wide scheduling. The exceptions for scientific research in the NDPS Act:

- Central Government to take measures for preventing and combating abuse of and illicit traffic in narcotic drugs, etc.—(*1*) Subject to the provisions of this Act, the Central Government shall take all such measures as it deems necessary or expedient for the purpose of preventing and combating abuse of narcotic drugs and psychotropic substances and the illicit traffic therein^3^ (and for ensuring their medical and scientific use).
- (2) In particular and without prejudice to the generality of the provisions of sub-section (1), the measures which the Central Government may take under that sub-section include measures with respect to all or any of the following matters, namely:

…(*d*) identification, treatment, education, after care, rehabilitation and social re-integration of addicts^4^; [(*da*) availability of narcotic drugs and psychotropic substances for medical and scientific use] [(9) (Ch. 2, Sec. 4)].

Exceptions for scientific research in the NDPS Act as stated in RCS Order 2013:

No person shall manufacture, distribute, sell, purchase, possess, store, or consume any controlled substance included in Schedule-A without unique registration number in Form-A issued by the Zonal Director of Narcotics Control Bureau:…Provided further that the Government or autonomous institutions, Schools or Colleges or Universities recognized by the Government, registered Scientific Societies and Hospitals using any controlled substance in Schedule-A for educational, scientific and analytical purposes are exempted from the registration [(9) (Sec. 4.1)].

The ability to research fentanyl and FRS is particularly sensitive in India, as fentanyl is included in India's list of “essential narcotics drugs” (ENDs). The NDPS Act was amended in 2014 to distinguish a class of substances, ENDs, for medicinal pain management. This amendment transferred the power to designate, manufacture, and distribute ENDs from the state governments to the central government, unifying the distribution of these substances at the central level. The power of ENDs legislation rests with the State Drug Controller, an agency that determines which medical establishments, known as recognized medical institutions (RMI), receive ENDs, and it oversees their dispensation (88). Six substances make up these ENDs: fentanyl, morphine, codeine, hydrocodone, oxycodone, and methadone (89). The S.O.1181(E) notification in 2015 places fentanyl among five other ENDs, “The Central Government hereby notifies for medical and scientific use, the following narcotic drugs to be essential narcotic drugs, namely:…(2) 1-phenethyl-4-N—propionylanilino-piperidine (the international-non-proprietary name of which is Fentanyl) and its salts and preparations, admixtures, extracts or other substances containing any of these drugs…” (89).

Class-wide control over fentanyl may complicate certain aspects of ENDs research, thus, a more streamlined approach to permit research may be necessary. A balance should be reached to protect the public by minimizing the illicit manufacture and distribution of fentanyl/fentanyl analogs while allowing for scientific research with as few controls and roadblocks as possible. Class-wide bans will likely encompass inconveniences for universities, research institutes, chemical companies, and other legitimate entities, though, the potential to reduce future damage to society from this legislation, vs. relatively minor inconveniences to researchers, should put these considerations into perspective.

## Conclusion

The Indian government should ensure that opioids are properly prescribed and supervised to reduce misuse, addiction, and overdoses. Patients receiving opioids for pain management should be screened, prescribed quantities corresponding only to their medical needs, and monitored for evidence of addiction. Most regions in India maintain punitive, rather than harm reducing, policies for people with drug dependence (90). The Ministry of Social Justice and Empowerment oversees the reduction of demand for illicit substances in India and should increase funding for opioid substitution therapy (OST), evidence-based treatment, fentanyl test strip and naloxone distribution (from healthcare facilities), pre-exposure prophylaxis (PrEP), which have been shown to reduce HIV transmission among drug users (91), safe needle exchange programs, and de-stigmatization drives. Young-adult male PWID in India have a heightened risk for needle sharing and sex with multiple partners, thus, tailored interventions uniquely targeting younger generations should be instituted (80), as younger and older generations of PWID may respond differently to different treatments. When new harm reduction programs are implemented, well-trained and non-judgmental staff, as there is evidence of judgmental attitudes among harm reduction service employees in the region (92), are necessary.

In 2019, India amended its drug laws to increase control over the online distribution of precursor chemicals (93). Operators of online markets in India are required to detail, track, and surrender information concerning the transactions of controlled precursors. These illicit transactions are typically conducted on the “darknet,” which maintains online markets usually requiring passwords or additional software to access and are detached from search engines. Large quantities of IMF and FRS are distributed globally through the darknet (94), and this method is becoming increasingly popular in India. For example, in 2017 Indian officials arrested 15 people operating two illegal pharmacies distributing large quantities of narcotics online (95). Online markets have been the central means for Chinese distributors to the sell IMF and FRS to Mexican cartels and U.S. buyers for nearly a decade (44), and this will likely be the dominant platform for Indian dealers as well. Indian authorities should actively pursue online distributors by posing as buyers, tracking sellers, and ultimately locating, prosecuting, and dismantling the manufacturers.

The scale of the Indian chemical, pharmaceutical, and medical industries enable illicit activity to occur nearly unchecked. For example, India is the world's largest legal manufacturer of the fentanyl precursor NPP, which, as per international treaties of which India is a party (54, 56, 96), should undergo strict supervision in both manufacture and distribution. Though, distribution mismanagement has been problematic, with shipments of NPP sent to questionable and unverifiable destinations (93). In 2019, the International Narcotics Control Board (INCB), which is the supervisory organ of the UNODC, investigated India's distribution of NPP and found that some international sales were being conducted without informing the appropriate regulatory agencies (93), and, on top of this, the investigation was unable to determine how some shipments of NPP were used by certain buyers. Indian chemical and pharmaceutical companies should undergo regular and unannounced inspections by experts accompanied by international observers. India's chemical industry is too large and complex for status quo enforcement, and these problems will worsen when attempting to enforce bans on greater numbers of FRS.

The Indian government should form strong partnerships with China, Pakistan, and the U.S. to share narcotics intelligence and advanced fentanyl detection technologies. For example, the 2018 arrest of three men in Indore, India, with 10 kg of fentanyl was a product of U.S. DEA intelligence and Indian cooperation (2). These cooperating states should closely monitor India's import of fentanyl precursors from China, and, for fentanyl synthesized in India, its Indian and Indian-Chinese export channels (as Chinese TCOs have been connected to Indian fentanyl production and have a long history of entrenched involvement in the South and Southeast Asian drug trade (2).

As a major supplier of FRS to India and an influential regional actor, China should be a key partner in India's international efforts to control synthetic opioids. Diplomatic setbacks in 2022 have disrupted U.S. and Chinese cooperation to combat fentanyl production and distribution (97), providing India the opportunity to bridge this diplomatic impasse and bring the three countries together in a common cause. Collaboration should also involve Pakistan, as Pakistani militants routinely distribute opioids to a large population of Indian users, particularly in the Kashmir and Himalayan regions (98). Moreover, around 45 percent of all opioids exported from Afghanistan first stop in Pakistan (12), where it is redistributed to international destinations, India included (99), posing a serious threat to Indian public health. Sharma (100) explains, “A portion of the drugs coming from Pakistan are sold in Kashmir, and the rest is transported into other parts of India” (para. 9).

U.S., Chinese, and Pakistani intelligence services, working independently or in tandem, should cooperate with India's Ministry of Home and Defense (specifically the Narcotics Control Bureau and regional police), the Ministry of Health and Family Welfare (specifically the Drugs Controller General of India), and the Ministry of Finance (specifically the Central Bureau of Narcotics, Customs, and Excise), along with other regional and state authorities. Cooperation with several different Indian agencies with different levels of organization and efficiency, who themselves often fail to adequately cooperate with each other due to overlapping responsibilities and chaotic administration (101), presents coordination challenges. Additionally, Indian legislation governing IMF, FRS, and other narcotics is complex, providing extensive room for different ministries and agencies to misinterpret laws and error in enforcement efforts and international cooperation. India should reevaluate its drug control network to simplify and streamline interactions and communications with the U.S., PRC, Pakistan, and international drug control regimes. The capacity for international actors to influence Indian exports of IMF and FRS will be limited if they are subject to complex, chaotic, and corrupt Indian bureaucracy. India should also uphold its obligation to maintain the narcotics laws, policy, and international cooperation advanced by the United Nations international drug control conventions of 1961, 1971, and 1988, of which India is a signatory (96).

Cooperation between India, China, Pakistan, the U.S. and other regional actors should include intelligence sharing, law enforcement and military partnerships on the ground level, and the sharing of responsibilities for monitoring points of exit. Advanced technologies to combat fentanyl production and distribution should also be shared among concerned parties, specifically cutting-edge IMF and FRS detection methods. Advanced fentanyl analog detection methods are necessary. Wharton et al. (102) tested 19 commercially available immunoassay fentanyl/fentanyl analog test kits and found the effective detection of targeted fentanyl analogs and other closely related fentanyl analogs, though certain fentanyl analogs, such as 4-methoxy-butyryl fentanyl and 3-methylfentanyl, had lower rates of detection. Liquid chromatography-high resolution mass spectrometry (LC-HRMS) has shown promise in fentanyl analog detection, effectively detecting various fentanyl/fentanyl analogs among post-mortem biological samples, particularly fentanyl and the fentanyl analogs carfentanil, 4-fluorobutyrylfentanyl, and butyrylfentanyl (103). A similar method employing software-assisted data mining technologies has also effectively detected the fentanyl analog phenylfentanyl and fentanyl metabolites such as 4-ANPP (104). Improved fentanyl analog detection methods and technologies should be prioritized, as new and dangerous fentanyl analogs are regularly introduced to illicit drug markets and require swift and effective detection. Innovative fentanyl/fentanyl analog detection methods and technologies should be shared among law enforcement agencies in cooperating states.

Blanket ban legislation covering fentanyl and its precursors and strict enforcement implemented by Indian authorities is necessary. Greater legislative control and vigorous enforcement, specifically at ports of entry (POE), as many Chinese made precursors are imported *via* express consignment carrier (ECC) (105), should disrupt IMF and FRS production and distribution. The U.N. international drug control conventions of 1961, 1971, and 1988 also operate without class-wide controls for fentanyl and its precursors. Amending these agreements would be thorny, hence, a complementary and binding international treaty dedicated to the class-wide scheduling of fentanyl and its precursors, of which India would likely be a key participant, should also be forwarded (106).

## Author contributions

CW: supervision, project administration, validation, writing, review, editing, and funding acquisition. NL: conceptualization, investigation, writing, review, and editing. All authors contributed to the article and approved the submitted version.

## Funding

The Fundamental Research Funds for the Central Universities, and the Cross-Disciplinary Research Project of Zhejiang University, project number: 2022-502110-0003.

## Conflict of interest

The authors declare that the research was conducted in the absence of any commercial or financial relationships that could be construed as a potential conflict of interest.

## Publisher's note

All claims expressed in this article are solely those of the authors and do not necessarily represent those of their affiliated organizations, or those of the publisher, the editors and the reviewers. Any product that may be evaluated in this article, or claim that may be made by its manufacturer, is not guaranteed or endorsed by the publisher.

## Footnotes

^1^The U.S. controlled NPP as a Schedule I substance in 2008 (73 FR 43355).

^2^Armstrong et al. (79) also found that young-adult male PWID in northeast India engaged in more risky injecting compared to older male PWID, though, unprotected sex was less prevalent among young-adult male PWID.

^3^Ins. by Act 16 of 2014, s. 3 (w.e.f. 1-5-2014).

^4^Ins. by Act 16 of 2014, s. 3 (w.e.f. 1-5-2014).
